# The Effects of Arsenic Exposure on Neurological and Cognitive Dysfunction in Human and Rodent Studies: A Review

**DOI:** 10.1007/s40572-014-0012-1

**Published:** 2014-03-21

**Authors:** Christina R. Tyler, Andrea M. Allan

**Affiliations:** Department of Neuroscience, University of New Mexico School of Medicine, Albuquerque, NM USA

**Keywords:** Arsenic, Exposure, Arsenite, Arsenate, Teratogen, Development, Neurotoxicity, Cognition, Alzheimer’s disease, Cholinergic, Monoaminergic, Glutamatergic, Neurogenesis, Children, Adult, HPA, Cortisol, Glucocorticoid receptor, IQ, Neurological deficits, Mood disorders, Epigenetics, Histone modifications, DNA methylation, Depression, Anxiety, Lead

## Abstract

Arsenic toxicity is a worldwide health concern as several millions of people are exposed to this toxicant via drinking water, and exposure affects almost every organ system in the body including the brain. Recent studies have shown that even low concentrations of arsenic impair neurological function, particularly in children. This review will focus on the current epidemiological evidence of arsenic neurotoxicity in children and adults, with emphasis on cognitive dysfunction, including learning and memory deficits and mood disorders. We provide a cohesive synthesis of the animal studies that have focused on neural mechanisms of dysfunction after arsenic exposure including altered epigenetics; hippocampal function; glucocorticoid and hypothalamus-pituitary-adrenal axis (HPA) pathway signaling; glutamatergic, cholinergic and monoaminergic signaling; adult neurogenesis; and increased Alzheimer’s-associated pathologies. Finally, we briefly discuss new studies focusing on therapeutic strategies to combat arsenic toxicity including the use of selenium and zinc.

## Introduction

Arsenic is ranked first among toxicants posing a significant potential threat to human health based on known or suspected toxicity [1]. This naturally occurring metalloid is a known poison, a co-carcinogen, and in lower concentrations has been shown to increase susceptibility to cognitive dysfunction [2]. Currently, the permitted concentration of arsenic in water is 10 μg/L (10 ppb). Yet, an estimated 100 million people worldwide are exposed to excessive amounts of arsenic via drinking water (in the ppm range). Many of these individuals obtain drinking water from unregulated sources (wells) or live in regions where arsenic levels are high, as in Bangladesh. As arsenic leaches from rock formations into water sources as the water table recedes, exposure to high amounts of arsenic will continue to persist as the demand for clean water increases. This phenomenon particularly affects the Western region of the United States, where it is estimated that certain areas contain up to 3100 μg/L arsenic (31 ppm) in drinking water, on par with levels reported in Taiwan and China [2]. While the World Health Organization (WHO) and the Environmental Protection Agency (EPA) regulate water sources of arsenic, lack of strict regulations on food, beverages, and air quality can lead to increased arsenic exposure [3]. Ingestion of arsenic activates metabolic pathways for excretion, resulting in a number of metabolites, some of which are more potent and toxic than the originally ingested inorganic form of arsenic (iAs), including mono- and dimethylated arsenicals [4–8].

All forms of arsenic, including inorganic and methylated arsenicals, accumulate in many parts of the brain, with the highest accumulation in the pituitary [9]. Additionally, arsenic is a well-established teratogen that crosses the placenta during development, and in high concentrations, induces growth delays and neural tube defects [10–12]. Epidemiological studies reviewed in this paper have investigated the neurological and cognitive effects of arsenic on children and adults. Evidence presented suggests that chronic ingestion of arsenic alters a number of intelligence measures and induces learning deficits and mood disorders like depression. Information on dose, concentration, extent, and method of exposure of arsenic will be given for each study. We provide a detailed review of the mechanisms involved in arsenic-induced toxicity in the brain. These include altered glucocorticoid signaling, cholinergic and monoaminergic signaling, adult neurogenesis and other forms of synaptic plasticity, and behavioral deficits including learning, memory, and locomotion. The last section of this review discusses new studies on therapeutics used in rodent models that may eventually be useful for remedying arsenic-induced neurological deficits in human populations.

## Epidemiological Studies

### Children Exposed to Arsenic: Cognitive Assessments

Over the past two decades, various epidemiological reports have shown that arsenic exposure may alter cognitive function, particularly learning and memory during childhood. A recently published meta-analysis focused on the impact of arsenic exposure on intelligence measured by IQ tests [13••]. Researchers concluded that arsenic exposure was associated with a 0.4 decrease in IQ in exposed children [13••]. This appears to be a minor decrease in IQ, but these changes could have cumulative effects later on in life. For our purposes, we will provide a synopsis of epidemiological studies focusing on other neurobehavioral measures in children.

Several studies have shown a relationship between low arsenic exposure (in the parts-per-billion range) and children’s intellectual performance on a battery of cognitive tests that cover a number of skills and processes. For example, a 2007 study found a significant association between urinary arsenic concentrations greater than 50 μg/L and poor scores on tests measuring visual-spatial reasoning, language and vocabulary, memory, intelligence, and math skills in 6-8 year old (y.o.) children from Mexico [14]. It should be noted that researchers found a sex-specific effect of arsenic: males performed more poorly on all assessments compared to females. However, males had higher arsenic concentrations (mean 11.85 μg/L) in their urine, which could account for these differences. In another cohort of children in Mexico (6-7 y.o. with 55 μg/L urinary arsenic), researchers reported poorer scores in arsenic-exposed children on measures of language and vocabulary and a modest association with hyperactive behavior using the attention deficit hyperactive disorder (ADHD) index [15]. In India, a cross sectional study on 351 children (5-15 y.o.) exposed to an average of 147 μg/L arsenic in water throughout development and childhood found an association between arsenic exposure and poor performance in several measures focusing on vocabulary, math skills, memory, and overall cognition; however, confidence intervals and the age range of participants in this study were broad [16]. When accounting for age range and other covariates (education, levels of arsenic, socioeconomic status), there was a dose-dependent relationship between arsenic levels in water and poor performance scores on intelligence measures [17]: children exposed to >50 μg/L arsenic had poorer intellectual performance than those exposed to <5.5 μg/L arsenic [17]. While low exposures to arsenic (< 2 μg/L) resulted in no measurable effects on IQ, moderate exposures (142 μg/L and 190 μg/L) resulted in decreases of 5 and 10 IQ points, respectively [18]. These findings, along with other studies (Table 1) support the association between moderate arsenic exposure and deficits in cognitive skills in children exposed during development.

**Table 1:** Impacts of arsenic on cognition in children and adults

Cognitive assessment	Exposure	Age	Finding	Reference
Intelligence (IQ)	Low and High	Children	↓verbal IQ ↓total IQ	Calderon et al. 2001 [42]
	Medium	Children	↓total IQ	Rosado et al. 2007 [14]
	Low and High	Children (5-15 y.o.)	↓total IQ	Von Ehrenstein et al. 2007 [16]
	Low	Children (9-10 y.o.)	↓total IQ	Wasserman et al. 2004 [17]
	Low	Children (6 y.o. and 10 y.o.)	↓total IQ	Wasserman et al. 2007 [30]
	Low and Medium	Children (5 y.o.)	↓total IQ (females) ↓verbal IQ (females)	Hamandani et al. 2011 [26•]
Cognitive skills (reading, writing, vocabulary, math)	Medium	Children (6-7 y.o.)	↓capacity in figure design, vocabulary, letter sequencing	Rosado et al. 2007 [14]
	Medium	Children (6-7 y.o.)	↓capacity in vocabulary	Roy et al. 2011 [15]
	Low and High	Children (5-15 y.o.)	↓capacity in vocabulary, language	Von Ehrenstein et al. 2007 [16]
	Low	Adults	↓capacity in executive function, mental acuity, verbal skills	O’Bryant et al. 2011 [43••]
Visual perception	Medium	Children (6-7 y.o.)	↓capacity in visual search	Rosado et al. 2007 [14]
	Low and High	Children (5-15 y.o.)	↓capacity in picture completion, object assembly	Von Ehrenstein et al. 2007 [16]
Mental health	Medium	Children	↑risk for ADHD	Roy et al. 2011 [15]
	Low	Adults	↑incidence of depression	Zierold et al. 2004 [53]
	Medium	Adults	↑symptoms of anxiety	Dang et al. 2008 [50], Dang et al. 2009
	Low → High	Adults	↓quality of life and mental health	Syed et al. 2012 [47]
	High	Adults	↑symptoms of altered mental health	Fujino et al. 2004 [51]
	Low → High	Adults	↑insomnia ↓general health	Guo et al. 2007 [49]
	Low → High	Adults	↑risk of psychiatric disorder, depression, anxiety	Sen et al. 2012 [52]

Exposure

Low: less than 50 μg/L (ppb) urinary arsenic or water arsenic

Medium: between 50 μg/L (ppb) and 100 μg/L (ppb) urinary or water arsenic

High: more than 100 μg/L (ppb) urinary arsenic or water arsenic

As mentioned above, a common finding in these studies seems to be the differential effect of arsenic among young males and females; while this has not received much attention [19], some research suggests that arsenic impedes growth and development in young females more than males, which in turn could impact cognitive function [20]. In studies where urinary arsenic levels between males and females are comparable, females aged 5-15 y.o. had a larger negative response to arsenic [21]. Overall, in addition to poor cognitive assessments, children exposed to arsenic in well water also have poor development scores (i.e., height), which correlates with lower intellectual function; this effect may be more pronounced in females than males [22].

#### In Utero and Cumulative Exposure to Arsenic

Most epidemiological studies focusing on the effects of arsenic exposure in children, with exposures occurring across several years, have reported impaired cognition regardless of the concentration of arsenic. Conversely, studies evaluating the impact of arsenic exposure only in utero fail to show consistent cognitive deficits. Analysis of arsenic in cord blood one day after birth (1.33 μg/L) and subsequent behavioral assessment (Brazelton Scale) showed an inverse correlation between arsenic levels and neurodevelopment in newborns in Nepal [23]. Yet, an early-life longitudinal study of pre- and postnatal arsenic exposure reported no significant correlations between maternal or child arsenic levels and psychomotor and mental development index assessments, behavior, or the maternal report of language acquisition in 18 month old children [24]. Additionally, infants born to mothers with urinary arsenic levels of 80 μg/L during pregnancy showed no cognitive impairment when accounting for age, sex, and socioeconomic status [25]. Measurable impaired cognition becomes evident later in life: a Bangladesh study from the same group demonstrated a correlation between the mother’s and child’s urinary arsenic content (80 and 51 μg/L respectively) and verbal IQ and full scale IQ but only in females at 5 years of age [26•]. Research focused on the adolescent time period of development provides more insight, suggesting that long-term cumulative exposure is more harmful for cognitive function including alterations in pattern memory, attention set shifting [27], full scale IQ, verbal IQ, and functional memory [28]. The levels of arsenic among these different cohorts of teens were highly variable, ranging from 17.8 μg/L as assessed in hair [28] to 132 and 185 μg/L arsenic in well water [27]; thus, conclusions determined from comparing these studies are limited. Additionally, data from toxin assessment in hair has been deemed somewhat insufficient to predict health outcomes as information of toxin content varies according to gender, ethnicity, and pharmacokinetics [29]. However, all groups in these studies had developmental exposure to arsenic. As with other teratogens, the timing, dose, and duration of arsenic exposure seem to determine the extent of neurological insults on children, and these insults may occur during development to produce cognitive dysfunction in adolescence or adulthood.

Chronic moderate exposure to arsenic, more so than high acute exposure, may induce greater harm on intellectual function in children. Several investigations by Wasserman and colleagues showed that the relationship between arsenic exposure and cognition in children might be age dependent. In a study of arsenic-exposed children aged 6 y.o., researchers found a negative association between arsenic and performance on the Wechsler Preschool and Primary Scale of Intelligence (WPPSI-III) [30]; yet this association was not as substantive as the inverse association observed in 10 y.o. children, who had longer arsenic exposure [17]. Both cohorts of primary school children showed reduced intellectual function and performance, reduced processing speed, and lower full-scale raw scores with arsenic exposures between 5-50 μg/L during development. Yet, the chronic nature of the exposure combined with the concentration of arsenic had the most adverse effect on cognitive ability and neurological functioning in children.

#### Factors Comorbid with Arsenic Exposure

One difficulty in drawing conclusions of the effect of arsenic on cognitive ability is that exposure is commonly associated with other factors that could affect the outcomes of these studies (Table 2). These include exposures to a mixture of metals, low socioeconomic status, and poor nutrition.

**Table 2:** Factors comorbid with arsenic exposure

Exposure to arsenic impacts performance on a variety of cognitive assessments in children; however, other factors could contribute to the negative effects of arsenic.
Metal mixtures: Arsenic is not the only naturally occurring element that leaches into water sources. Other metals including lead, mercury, manganese, cadmium, and copper exist in significant quantities, particularly in well water. Thus, arsenic may not be acting alone in disrupting cognition in children; it may be the mixture of metals that is reducing intelligence and other measures of neurological function.
Socioeconomic status: Unfortunately, millions of children live in poverty all over the world; and low socioeconomic status correlates with reduced cognitive function. Children living in poor areas typically do not have access to clean water and are more likely to be exposed to high levels of arsenic.
Nutrition: Along with lower SES and exposure to metals, children exposed to arsenic typically have poor nutrition, likely due to an impoverished situation. Additionally, metabolism of arsenic can interfere with nutrient absorbance. It’s likely that arsenic exposure in combination with these other factors accounts for the cognitive deficits observed in exposed children.

#### Metal Mixtures

The presence of arsenic in water or the environment does not preclude the presence of other elements; in fact, it is probable that a number of metals (Pb, Mn, Cd, Hg) are comorbid in individuals with arsenic exposure in the studies provided here. Reports on developmental exposures to heavy metal mixtures suggest that combined exposure is associated with greater risk for cognitive dysfunction, including behavior and impaired neurological (CNS) development [31]. The incidence of mental retardation is highly correlated with the presence of soil metals in rural areas (As, Cu, Pb, Mn, Hg), and the probability of intellectual disability in children increases as the concentration of arsenic and lead in the soil increases [32, 33]. Yet, determining the effect of one metal in isolation is quite difficult. In several studies, the concentration of lead in blood, urine, or water (while low), still positively correlates with arsenic levels [23]. Lead in particular is a known toxicant imparting severe neurotoxic effects on the brain especially in children. Of the studies described here, only a few discuss comorbid exposures and provide information about other metal concentrations. Poor scores on cognitive assessments have been reported in children exposed to both arsenic (slightly above and below the 10 μg/L EPA standard) and manganese (500 μg/L) in well water, though the arsenic and manganese interaction was not significant [34]. Arsenic, in particular, was associated with poor working memory and lower verbal comprehension scores as previously reported in a smaller prospective study ([34]), although a significant manganese arsenic interaction was found in this study [28]. As such, further investigation on developmental neurotoxicology of arsenic in combination with other substances is warranted. The possibility that observations in epidemiological studies may be induced by comorbid factors in addition to arsenic cannot be ignored.

#### Socioeconomic Status

Overall, arsenic exposure is correlated with lower socioeconomic status (SES) [14], and SES correlates with lower measures on tests assessing infant development and cognition [25]. The effects of poverty and parental stress due to low SES are significant risk factors in neurodevelopment disorders [35], and parsing out these effects from arsenic exposure is quite difficult. Additionally, control cases used for epidemiological work are typically from more affluent areas without arsenic exposure. In China, cross-sectional analyses have shown that children who live in rural areas have lower IQ scores than children who live in urban areas [18, 21, 36]. Children residing in poor rural areas are more often exposed to arsenic, which may exaggerate the inverse associations found between arsenic in drinking water and IQ scores [37]. Low SES is often associated with less education, especially in developing countries; one study reported that cognitive ability in children exposed to arsenic increased as maternal education increased from primary to secondary school [16]. However, many epidemiological studies that controlled for education as a covariate still reported significant neurodevelopmental deficits imparted after arsenic exposure [23].

#### Nutrition

Malnutrition can have a substantial negative impact on cognitive development and performance even in the absence of arsenic or metal exposure. Children lacking proper nutrition during development, such as iron deficiency, have deficits in neurological function, including cognition [38]. Arsenic exposure is thought to increase the risk for anemia, which is a potential risk factor in blunted growth and development [39]. Current research has been focused on the possible connection between the lack of proper nutrients in diet during development (methionine, cytosine, protein, folic acid, vitamin B-12, choline, and betaine) and developmental delay [40, 41]. These nutrients are important as they boost the body’s methyl donor availability, which is critical in the metabolism of arsenic. Increased consumption of methionine, cysteine, and protein aids in the methylation of arsenic, thus enhancing excretion in individuals exposed to arsenic [41]. The ability of the developing body to metabolize arsenic and to properly execute epigenetic modifications could be impaired by the accumulation of arsenic. As such, understanding the nutritional status of the children in these studies may be critical to interpreting the effects of arsenic on cognition.

#### Summary of Epidemiologic Studies in Children

Overall, we can ascertain certain patterns from the epidemiological literature on the association between arsenic exposure and cognitive performance in children. A number of studies have shown that arsenic induces cognitive deficits in children, even at low concentrations. Arsenic water levels or urinary arsenic levels correlate with poorer performance and scores on intelligence measures, and verbal IQ seems to be the most affected cognitive skill. These effects persist into adolescence, such that cumulative arsenic intake may be a greater risk factor than acute intake in cognitive dysfunction. Higher concentrations of arsenic exposure can alter growth and development in children, leading to neurological deficits, and females seem to be at greater risk than males, although few studies have systematically evaluated sex differences. Potentially confounding factors in these studies include the comorbidity of other metals with arsenic exposure, the correlation between high arsenic exposure and poor socioeconomic status, and poor nutrition in children exposed to arsenic [42]. Studies on cohorts from around the world, including China, the United States, Taiwan, Bangladesh, and Mexico, have all provided significant research supporting the negative effects of arsenic on childhood development and cognitive ability.

### Adults Exposed to Arsenic: Cognitive Assessments

Most epidemiological research has focused on cognition in children, and reports on the effect of arsenic exposure on adult cognition are limited. However, a series of studies has recently revealed a significant correlation between arsenic exposure and altered adult cognition, particularly for symptoms associated with Alzheimer’s disease. The FRONTIER project is an ongoing cohort study focused on a group of West Texas residents in an area that contains arsenic levels close to the EPA standard in groundwater (3-15 μg/L). Long-term and continuing chronic exposure to low levels of arsenic via drinking water significantly correlated with poorer scores in tests examining language, visual and spatial skills, and executive function, all of which indicate cognitive dysfunction [43••]. Poorer scores in global cognition, processing speed, and immediate memory were also found; these particular deficits have been associated with Alzheimer’s disease [43••, 44•]. Individuals exposed to 10.6 μg/L arsenic scored significantly worse on global cognitive assessment compared to those exposed to 6.5 μg/L arsenic, suggesting that 10 μg/L EPA standard may not be sufficient to prevent arsenic-induced cognitive deficits [44•].

There are several caveats to the FRONTIER studies. The Mini-Mental State Exam (MMSE) used for cognitive assessment has been criticized for its bias against less educated participants [45, 46]; indeed, FRONTIER participants in the 10.6 μg/L group did have less education than controls [44•]. Additionally, most of these assessments rely on verbal responses, reading, and writing, while other tests require proper auditory perception and visual acuity. The average age of the 434 FRONTIER participants in these studies was 62 years old, thus if arsenic exposure affected sensory, perception, or communication, participants may perform poorly even if no cognitive impairment exists. Finally, the arsenic concentrations in both of these FRONTIER studies were determined using the ArcGIS program based on ground water measurements made by the Texas Water Development Board; direct measurement of arsenic was not performed. However, despite these limitations, the findings presented here are important in supporting that cumulative, long-term exposure to low levels of arsenic, levels considered safe by the EPA, can impart cognitive deficits in adults.

### Adults Exposed to Arsenic: Psychological Assessments

While the number of studies concerning cognitive and mental disorders after toxic exposures has increased over the past ten years, specific characterization concerning arsenic exposure and psychological health requires more epidemiological investigation. Of the studies presented here, several point to a strong correlation between arsenic toxicity and mental health status. These data are reported from all over the world including China, Bangladesh, India, and the United States. Altered cognition is typically concurrent with disturbances in mental health. As such, it is difficult to assess the validity of studies primarily focused on psychological health in arsenic-affected individuals, as the previous data we have presented shows a strong correlation between arsenic exposure and cognitive deficits. However, despite the overlap between psychological and cognitive disorders, there is substantial evidence to suggest that arsenic exposure increases the risk of impaired cognition and enhanced susceptibility for mood disorders.

#### Bangladesh

Bangladesh has one of the highest reported arsenic exposure rates in the world, and yet, few studies have explored the relationship between arsenic exposure and mental health. A recent cross-sectional study, published in 2012, found that patients with arsenic poisoning reported a lower quality of life and scored lower on a validated mental health index compared to individuals without arsenic exposure [47]. Arsenic poisoning has strong societal implications in Bangladesh, often resulting in individuals with arsenic exposure ostracized from the community [47]. This lack of social acceptance could contribute to the lower quality of life and mental health scores in these individuals; however, other studies on the effects of arsenic and mental health concur with those reported in this study.

#### China

China accounts for approximately half of the world’s arsenic production and consequently its pollution [48], and there are many regions that contain naturally high amounts of arsenic in soil deposits. Arsenic toxicity studies from China typically focus on symptoms of neurotoxicity, including loss of hearing, loss of taste, blurred vision, and tingling/numbness in the limbs [49]. Altered mental health is comorbid with these ailments, including a prevalence of insomnia [49], anxiety and depression [50], and symptoms of distress [51]. Assessments of mental health were congruent with education levels of the participants; however, as described above, exposure to metal mixtures is common, and participants exposed to metals in one study showed significantly greater urine and hair concentrations of arsenic, lead, and cadmium than control participants [50]. Additionally, water in these villages contained between 15-1860 μg/L arsenic, much higher than the Chinese Drinking Water Standard of 50 μg/L.

#### India

In India, over 1.5 million people have been exposed to high levels of arsenic with more than 200,000 cases of arsenicosis. Cross-sectional analysis of over 1169 arsenicosis patients between the ages of 18-65 y.o. revealed that 19 % of patients developed some type of psychiatric disorder, compared to an average 7 % prevalence of mental disorders in India [69]. Of the 19 % of patients, most were categorized as having depression and/or anxiety; yet, the prevalence of depression in India is only 3.7 %. The participants in this study were from seven different villages, 90 % of which had arsenic levels ranging from 25-900 μg/L. Most patients were males with low SES and a 25 % rate of unemployment. As noted in the studies from Bangladesh, it is difficult to determine if these patients exhibit depression due to the state of their overall health (arsenicosis) or if depression is a manifestation of the arsenic toxicity. However, in Indian society, arsenicosis patients are able to maintain their traditions, kinship, and cast relations to cope with stress of the disease [52]. Similar to observations of the general population, anxiety and depression were the most common psychiatric disorders in arsenicosis patients; however, lower SES, the diseases associated with arsenic poisoning, body image, and low self-esteem are predisposing factors of psychological issues in this population.

#### United States

The United States has more stringent standards of arsenic water quality than other countries (the EPA limit is 10 μg/L), yet this concentration may not be low enough as suggested by the literature on cognitive dysfunction. A 2004 cross-sectional study covered a wide range of locations in areas with low concentrations of arsenic and revealed a significant association between low arsenic exposure (2-10 μg/L) and poor mental health, particularly depression. Individuals with cumulative, long-term exposure to low concentrations of arsenic (2-10 μg/L) for more than 20 years were significantly more likely to exhibit depressive symptoms than those drinking less than 2 μg/L arsenic [53]. Interestingly, participants consuming more than 10 μg/L arsenic were also more likely to have had cardiac bypass surgery, high blood pressure, and circulatory issues than those drinking less than 2 μg/L arsenic in water [53]. Unlike studies from India, Bangladesh, and China where low levels of arsenic were not assessed, this study suggested that low exposure, under EPA limits of 10 μg/L, is associated with higher rates of adverse mental health. However, evaluating the validity of the conclusions from this study is difficult because of the lack of the critical methodological information, (e.g., participant health status, and the type and analysis of the assessment tools), which are all important in interpreting the findings.

#### Summary

There are several limitations to these studies, including some with small sample sizes, all with variable levels of arsenic toxicity, and arsenic-affected individuals typically living in rural areas and having lower SES. Additionally, assessment of dose-response relationships and mental health has not been determined: it would be useful to have high and moderate arsenic exposures compared in the same study. While it is difficult to disentangle the contribution of arsenic and that of general health in many of the studies conducted in countries outside of the United States, the findings presented thus far demonstrate a correlation between arsenic exposure, cognitive dysfunction, and mental health. There is clearly substantial evidence that arsenic exposure diminishes cognition and increases mood disorders in human populations. Animal and invertebrate studies have replicated many of these observations and aided in the search for the mechanisms for the adverse health impacts of arsenic.

## Mechanisms of Action

It is difficult to use the epidemiological literature to identify mechanisms, as long-term exposures to arsenic are likely compounded with exposures to pollution, poor diet, and low SES. Basic science research is poised to control for these confounding factors, including extent and timing of exposure. This research has determined that arsenic imparts its toxicity on the body via a number of mechanisms. These include the depletion of methyl groups affecting epigenetic profiles, the uncoupling of oxidative phosphorylation and increased reactive oxygen species, the inhibition of thiol-containing enzymes and proteins (including the depletion of glutathione), altered signal transduction and cell proliferation, and reduced DNA repair inducing genotoxicity [1, 6]. As these mechanisms have been discussed in detail in other reviews, we cover mechanisms particularly related to the brain. These include hippocampal dysfunction; glutamatergic, glucocorticoid, cholinergic, and monoaminergic signaling; pathways associated with Alzheimer’s disease; and synaptic plasticity, particularly neurogenesis. To begin, we briefly provide results from the few studies focusing on neural epigenetic patterns altered after arsenic exposure.

### Epigenetic Patterns

Arsenic accumulation and subsequent toxicity is likely mediated through multiple mechanisms of action. Of particular interest over the past few years is the impact of arsenic on the epigenome. While there have been several studies determining the impact of arsenic on epigenetic regulation in cancer cells, the liver, and other parts of the body, very few have focused the brain. Exposure to 3 and 36 ppm arsenic throughout gestation increased DNA methylation on two genes involved in neural plasticity in rat cortex and hippocampus at one month of age [54•]. Hypomethylation of these genes in both regions was observed after four months of cumulative exposure to arsenic; these animals also displayed deficits in fear memory as well, although the link between hypomethylation and memory was correlational. Since arsenic metabolism requires methyl groups derived from S-adenosylmethionine (SAM) for excretion, it is plausible that arsenic depletes SAM leading to alterations in DNA methylation. In vitro studies corroborate this assertion: 25 μM arsenic exposure for 24 hours depleted SAM concentrations, increased global DNA hypomethylation, and repressed *Dnmt1* and *Dnmt3a* expression [55]. Epidemiological work has shown that DNA methylation is affected by arsenic in human populations as well, including umbilical cord blood, but discussion of those studies is beyond the scope of this review [56–59]. We can determine that exposure to arsenic does induce epigenetic modifications to the DNA, which may result in aberrant gene expression even in the brain; however the link between DNA methylation on particular genes and cognitive deficits has yet to be elucidated.

Other epidemiological studies have determined the impact of arsenic ingestion on histone modifications. Arsenic compounds have been shown to alter gene expression and posttranslational modifications (PTM) of histones in vivo; interestingly, researchers found a differential effect of arsenic on global histone modifications among males and females [60]. In vitro assessment of low doses of arsenic on histone modifications has also been performed; however, detailed discussion of this research is beyond the scope of this review [60–62]. Rodent studies with prenatal exposure to 100 μg/L arsenic demonstrated reduced global acetylation on lysine 9 of histone 3 (H3K9ac) in the cortex and hippocampus of postnatal day (PND1) pups, which was correlated to altered learning in adulthood [63]. Exposure to 3 and 36 ppm arsenic throughout development up to four months reduced myelination (for which methylation is required) and dimethylation of arginine residues on histones [64]. This could result from the altered expression and function of epigenetic modifiers or transcription factors, as arsenic impacts zinc-finger protein expression and function [65].

These studies demonstrate that arsenic impacts DNA methylation and histone modifications and alters the enzymes responsible for regulating these modifications. The effects of arsenic on the epigenome are related to the dose and extent of arsenic exposure as seen before, but also type of histone methylation mark, gene, and sex. As such, in determining mechanisms of arsenic toxicity it will be important to control for these variables both in epidemiological and molecular studies in the future. Additionally, cumulative low-level exposure to arsenic likely occurs over generations. The literature suggests that females are differentially more affected by arsenic than males. Since females are the key source of transgenerational effects (3rd generation ova are exposed to the in utero environment), arsenic could be impacting transgenerational epigenetics, including the imprinting of genes (from both males and females). This area of research could provide an insight into the effects of arsenic on the brain and body, yet there are no published reports on arsenic exposure and transgenerational epigenetic mechanisms to date.

### The Hippocampus

Studies on the mechanisms of arsenic-induced toxicity have established that arsenic alters learning and memory in behavioral assays and impacts multiple neurobiological processes including those of neurogenesis and cholinergic, glutamatergic, and monoaminergic signaling pathways. Recent work using animal models has revealed potent alterations in hippocampal function, morphology, and signaling leading to altered cognitive behavior after arsenic exposure. While the exposure paradigms and concentrations of arsenic have been highly varied, the overall conclusions have been congruent between studies (see Table 3).

**Table 3:** The impact of arsenic on behavioral tasks in rodent studies

Type of cognition	Task	Exposure	Finding	Reference
Learning and memory	Morris water maze (MWM)	Adult, chronic, high	↓acquisition	Luo et al. 2009 [71]
		Adult, chronic, low	↓acquisition	Sharma & Sharma, 2013 [66]
	Fear/cued conditioning	Adult, acute, low	↓recall	Cronican et al. 2013 [63]
	8-way radial arm maze	Developmental, chronic, low	↓acquisition	Martinez-Finley et al. 2009 [67]
	Y-maze; MWM	Adult, chronic, high	↓acquisition	Jing et al. 2012 [68]
	Radial water maze	Adult, acute, low	↓acquisition	Cronican et al. 2013 [63]
	Novel object exploration	Developmental, chronic, low	↓performance	Martinez-Finley et al. 2009 [67]
		Adult, acute, low	↓performance	Cronican et al. 2013 [63]
Locomotion and motor	Startle/reflex response/ spontaneous alteration/ developmental battery tests	Developmental, low → high	↓coordination ↑response time to completion no change in low group	Luo et al. 2013 [86]
		Developmental, low → high	No change in either group	Gandhi et al. 2012 [87]
	Rotarod	Adult, subchronic, high	↓coordination ↑ataxia	Yadav et al. 2009 [85]
	Spontaneous locomotor activity	Adults, chronic, low → high	Dose-related ↑locomotion with low dose ↓locomotion with high dose ↑ locomotion females	Bardullas et al. 2009 [83]
		Adults, chronic, low → high	no change in low group ↓ locomotion	Rodriguez et al. 2010 [84]
	Total movement	Adult, chronic, low → high	↓ movement at high dose	Bardullas et al. 2009 [83]
		Adult, subchronic, high	↓ total movement	Yadav et al. 2009 [85]
		Adult, chronic, low → high	↓ total movement	Rodriguez et al. 2010 [84]
	Grip strength	Adult, subchronic, high	↓ strength	Yadav et al. 2009 [85]

Exposure type:

Developmental: in utero to postnatal exposure (maternal consumption of arsenic)

Adult exposure: from weaning up to death

Exposure level:

High exposure: ppm (mg/L)

Medium exposure: above 100 ppb (μg/L)

Low exposure: below 50 ppb (μg/L)

Low → high; multiple doses examined

Exposure paradigm:

Acute: less than two weeks

Subchronic: less than one month

Chronic: more than one month

In particular, behavioral studies using hippocampal-dependent tasks, including the Morris Water Maze and fear conditioning, have corroborated epidemiological evidence of reduced cognition observed in humans with arsenic exposure. Table 3 details the behavioral paradigms and arsenic exposures used in these studies. Results from this body of literature suggest that regardless of dose, timing, or extent of exposure, arsenic induces hippocampal-dependent behavioral deficits in rodent models, suggesting impaired spatial, working, long-term, and short-term memory [63, 66–68]. Interestingly, the effect extends to nonmammalian species as arsenic exposure in *Danio rerio* (zebrafish) induces deficits in long-term memory as well [69]. Several of these behavioral deficits can be ameliorated with treatment; for example, sodium butyrate, a histone deacetylase inhibitor, attenuated deficits seen in the radial arm maze after two weeks of arsenic exposure of 100 μg/L during adulthood [66]. More detail on therapeutic interventions after arsenic exposure will be discussed in later sections of this review. Overall, work with animal models has demonstrated that arsenic induces deficits in multiple learning paradigms, particularly those relying on proper hippocampal function. Yet, identifying the hippocampus as a sensitive area for arsenic’s effects does not necessarily identify a molecular target. Alterations in multiple signaling pathways are localized to the hippocampal formation, but it should be noted that mossy fiber terminals in the hippocampus contain substantial amounts of zinc. Thus, the sensitivity of the hippocampus may be due to the effect of arsenic on zinc either via displacement or substitution. To date, no studies investigating this mechanism have been published.

### Glutamatergic Signaling

Arsenic impacts the synaptic activity of neurons localized to the hippocampus. Slices obtained from young and adult rats exposed to 100 μM arsenite in vitro had reduced amplitudes of excitatory post synaptic potentials (EPSPs) in the Schaffer collateral/CA1 synapses. Exposure inhibited long-term potentiation (LTP), a form of synaptic plasticity, in hippocampal slices from adult but not young rats; however, this effect was reversible after 20 minutes of washout. Arsenic exposure did not impair paired-pulse facilitation, indicative of presynaptic activity, suggesting that acutely applied arsenic does not affect presynaptic neurotransmitter release [70]. However, components of NMDA receptors, specifically NR2A, were reduced after three months of arsenic exposure (2.72, 13.6, 68 mg/L arsenic) in mice. Additionally, these mice exhibited morphological changes in hippocampal neurons—reduced size with a condensed nucleus and cytoplasm—along with capillary edema and irregular vascular endothelial cell morphology [71]. Similar morphological changes in hippocampal neurons were observed after a 3-month exposure of 8.2 mg/kg/day arsenic; both the striatum and hippocampus contained abnormally myelinated nerve fibers, while the hippocampus contained reduced mossy fiber terminals [68]. Brief, two week exposure to 100 μM arsenic also altered expression levels of mRNA for synapse related genes, including increased *Grin1*, *Syn2*, and *Stx6* expression, similar to results from our studies [63, 72].

In addition to altered synaptic activity and synapse-related gene expression, arsenic has been shown to impart alterations in central pathways involved in mediating learning and memory in the hippocampus. Using a three-month exposure model (from weaning until four months), arsenic-exposed animals had decreased NR2A expression, PSD-95, and p-CAMKIIα in the hippocampus with concurrent increased SynGAP expression, a known negative regulator of the Ras-MAPK pathway [73]. Reduced p-ERK1/2 in the hippocampus was also observed in the arsenic-exposed animals. These findings corroborate our own work demonstrating reduced ERK2 in the hippocampus after perinatal exposure to arsenic [74]. Thus, the Ras-MAPK/ERK pathway appears to be sensitive to arsenic damage; interestingly, zinc has been shown to alter this pathway as well [75]. It is possible that arsenic is acting in a similar fashion. Overall, altered Ras-MAPK/ERK signaling, LTP, and synaptic regulation in the hippocampus could underlie behavioral deficits suggesting arsenic-induced alterations in long-term episodic memory, associative learning, spatial learning, and working memory.

### Glucocorticoid Signaling

In addition to altered hippocampal-dependent behaviors, depressive-like symptoms have been observed in arsenic-exposed mice. We have demonstrated increased immobility in the forced swim task and increased latency for escape in the learned helplessness task, along with increased plasma corticosterone levels [74]. Corticosterone (CORT), the rodent equivalent of cortisol, is a stress hormone that plays a role in mediating the effects of the hypothalamus-pituitary-adrenal (HPA) axis in response to “stressful” events ranging from fear to learning. In humans, significant alterations in the HPA axis have been connected to depressive-like symptoms, and patients with depression typically report stress as a major factor in the onset of their depression. Thus, decreased behavioral ability and increased depressive-symptoms in arsenic-exposed animals correlate with the epidemiological data on reduced cognition in humans; as such, arsenic may be mediating not only cognitive impairments but also mood disorders in humans via the glucocorticoid signaling pathway.

CORT signaling is mediated through the corticosterone receptors, specifically the mineralocorticoid (MR) and glucocorticoid (GR) receptors. When activated, MR and GR translocate to the nucleus to allow for binding to response elements (MRE or GRE) on genes for transcriptional activation or repression. CORT signaling, via its receptors in the hippocampus, is responsible for imparting an inhibitory tone for the HPA axis. Our work has demonstrated nuclear levels of GR are much lower in adult mice perinatally exposed to arsenic than levels observed in controls in hippocampal tissue [67]. Additionally, we have shown decreased GR binding to and expression of *H-Ras* and *Raf-1*, genes involved in modulating the MAPK pathway with GRE binding sites [76]. In vitro studies have demonstrated that various levels of arsenic impact GR-mediated transcription in a bidirectional manner: high arsenic levels (1-3 μM) induce an inhibitory effect, while low levels (0.05–1 μM) seem to produce a stimulatory effect [77]. Arsenic’s impact on GR is predicated on the DNA binding domain (DBD) within the GR. Other steroid receptors, including the MR and the progesterone receptor respond to arsenic in a similar biphasic manner as the GR [78]. Interestingly, arsenic’s effects on transcriptional regulation of the estrogen receptor seem to be only inhibitory [79]. A recent study predicted that the GR pathway was a common mediator of metal-induced birth defects: indeed, arsenic-induced deficits, specifically neurodevelopmental toxicity, were prevented by inhibition of GR signaling in a chick embryo model [80]. Oscillatory signaling of the HPA axis, in addition to proper GR localization for MAPK activation, is paramount for proper learning and memory. This signaling may be impaired in arsenic-exposed animals: while they have elevated circulating CORT, their ability to initiate a proper HPA response is blunted after a stressor [81•]. Thus, the susceptibility of the GR to arsenic may play an important role in hippocampal-related deficits, including reduced learning and increased depressive-like symptoms (observed in rodent models) and may underlie mood and cognitive deficits seen in human studies as well.

### Cholinergic Signaling

Motor learning, cholinergic signaling, and locomotion are all affected by arsenic exposure in rodent models. Early studies demonstrated impaired motor coordination and delayed spontaneous alteration in rats chronically consuming arsenic (36 mg/L) for four months [82]. However the increased locomotion reported in this study has been challenged by more recent work: while altered locomotion is a common behavior seen in arsenic-exposed mice, whether this behavior is hypo- or hyperlocomotion seems to be dependent on sex and the arsenic exposure paradigm (see Table 3 for studies). Low levels of arsenic exposure seem to induce hyperactivity in male mice, while high levels induce hypoactivity [83, 84]. Conversely, female mice consuming any level of arsenic display hyperlocomotion [83]. Subchronic exposures to arsenic increase locomotion but impair motor coordination in both sexes [85], but reports on in utero exposure are conflicting, with some suggesting impaired neural reflexes and others reporting no changes in locomotor behavior [86, 87]. A similar dose relationship between locomotion and arsenic level occurs in the zebrafish model, where moderate arsenic levels reduced locomotion (line crossing) and high levels increased the distance travelled [88]. Details on these studies, including dose, timing of exposure, and age of behavioral assessment are provided in Table 3. While parsing out verifiable locomotor actions from anxiety is difficult and likely to impact these studies, we can ascertain that high concentrations of arsenic induce hypolocomotion, moderate levels of arsenic induce hyperlocomotion, and low concentrations may induce no change in locomotion.

Altered motor coordination and locomotion could arise from abberant cholinergic functioning. Several reports have noted reduced acetylcholinesterase (AchE) activity and choline acetyltransferase (ChAT) functioning after arsenic exposure. Female rats exposed to 20 mg/kg arsenic for 28 days displayed deficits in the transfer latency of the passive avoidance response and decreased labeling of muscarinic cholinergic receptors in the hippocampus and frontal cortex [89]. These brain regions had reduced AchE activity and ChAT labeling after arsenic exposure as well. Interestingly, treatment with a bioactive component of the spice curcumin during arsenic exposure attenuated these observed effects in female rats [89]. Exposure to less arsenic (5 mg/kg body weight) also inhibited AchE activity in the brain and was associated with poorer performance in operant learning [90]; another study demonstrated that AchE activity decreased with increasing arsenic concentrations in male rats after five days of exposure [91]. Our analyses have shown that mRNA of *AchE* is increased in the adult dentate gyrus after developmental arsenic expoure to 50 μg/L arsenic, suggesting a compensatory mechanism for altered AchE activity [72]. These studies provide support for arsenic in the etiology of Alzheimer’s disease, as more reports confirm reduced AchE and ChAT along with degeneration of cholinergic neurons in AD pathology [92].

### Alzheimer’s Disease

Global cognitive impairments assessed after arsenic exposure have been associated with Alzheimer’s disease (AD) in the human population; for an in-depth assessment of the relationship between arsenic and Alzheimer’s disease, see [93]. For the purposes of this review, we will discuss arsenic exposure in the context of experimental studies. In vitro research using a cholinergic neuronal cell line has demonstrated that sodium arsenite and dimethylarsinic acid (DMA) have different effects on APP protein levels, β-amyloid formation, and altered activity of AchE and ChAT [94]. These findings corroborate our own: we have found altered mRNA expression of genes associated with AD, including *Appb1* and *ApoE*, and *Ache* in the dentate gyrus of adult male mice after developmental exposure to 50 μg/L arsenic [72].

For hallmark pathologies associated with AD, cell culture studies have demonstrated arsenic exposure increases β-amyloid protein and induces hyper-phosphorylation of tau protein, oxidative stress, inflammation, endothelial cell dysfunction, and angiogenesis [95]. Interestingly, zinc has also been shown to induce tau phosphorylation through an ERK-sensitive mechanism [96]; again, this effect may be due to a cation sensitive system (to zinc or arsenic) involving the Ras-MAPK/ERK pathway. In 2012, Piacentini and colleagues provided further evidence supporting the role of arsenic in the etiology of AD. The authors found a significant correlation between a genetic polymorphism of the protein glutathione S-transferase *GSTO1-1* in a population of 120 AD patients compared to 114 healthy controls (OR = 3.70); this transferase has been linked to both Alzheimer’s and Parkinson’s disease and is involved in arsenic metabolism, even in invertebrate systems [97–99]. While arsenic does not participate directly in REDOX reactions, it can induce oxidative stress via depletion of glutathione and impair REDOX reactions by inhibiting enzymes with sulfhydryl groups [100]; and oxidative damage is strongly associated with AD. In rodent models, arsenic exposure results in vascular endothelial dysfunction, impairment of learning and memory, altered nitrogen levels, and oxidative stress; all of which have been associated with symptoms of dementia [66]. Treatment with a histone deacetylase inhibitor, sodium butyrate, attenuated these cognitive and vascular deficits observed in arsenic-exposed animals [66]. Sodium butyrate has been used in the treatment of depression and improves cognitive skills in the Alzheimer’s mouse model (APP/PS1-21) [101]. Overall, we confidently state that arsenic imparts cognitive deficits associated with AD in rodent studies of exposure; however, very few epidemiological studies have investigated this relationship to date.

### Monoaminergic Signaling

Arsenic affects many transporter systems including the monoamines, dopamine (DA), serotonin (5-HT), and norepinephrine (NE). We have demonstrated that very low doses of arsenic (50 μg/L) during development increase 5-HT_1A_ receptors in the dorsal hippocampus in adult offspring [74]. Exposure to moderate levels of arsenic (1, 2, and 4 mg/L) in water for 60 days reduced levels of NE, DA, and 5-HT in both the cerebrum and cerebellum of 7-week old mice in a dose-dependent manner. mRNA levels of monoamine synthetases (including dopamine β-hydroxylase, tyrosine hydroxylase (TH), and tryptophan hydroxylase) were also all reduced after exposure to 4 mg/L arsenic [102]. Evidence from other studies using low to moderate doses of arsenic suggest differential effects on monoaminergic signaling based on sex, as observed in locomotion tasks. Four months of chronic exposure to 0.05, 0.5, 5.0, or 50 mg/L arsenic in drinking water decreased levels of DA in the striatum and hypothalamus in females (who also exhibited increased locomotion) but not in males [83]. *TH* and thioredoxin (*Trx-1 A*) levels were reduced in the striatum of males but not females; curiously, the opposite effect was observed in the nucleus accumbens where *TH* and *Trx-1A* were reduced in females but not males [83]. Conversely, in a different study on rats ingesting 50 mg/L arsenic in water, DA content in the striatum was increased in males, although no changes in DA, its metabolites, or serotonin were found in the prefrontal cortex or the nucleus accumbens [84]. This group also reported alterations in mRNA of several antioxidant genes including superoxide dismutase (SOD), *Trx-1*, and NE and DA receptor genes in arsenic-exposed animals dependent on dose and region of interest in the brain (nucleus accumbens, prefrontal cortex, or striatum). Upregulation of antioxidant factors (*Trx-1* and SOD) indicate compensatory mechanisms to overcome the oxidative byproduct effects of arsenic toxicity on thiol containing enzymes related to arsenic metabolism [84]. It is difficult to propose a mechanism of action for opposite effects based on sex for different brain regions. However, evidence from these studies, while conflicting, suggest that arsenic may induce a complex dose-response relationship, which may not be linear, on locomotion and the nigrostriatal dopaminergic system as seen in other studies.

High levels of arsenic exposure, which have been reviewed elsewhere, produce more definitive deficits on dopaminergic and serotonergic signaling in the corpus striatum, hippocampus, and frontal cortex [85, 103]. Interestingly, treatment with curcumin in these studies attenuates deficits in monoamines and increases nitric oxide (NO) and TH expression, in both sexes. More details on therapeutic strategies to combat arsenic toxicity will be provided later.

### Neurogenesis

Deficits in hippocampal-related behavioral tasks and increased depressive-like symptoms suggest that arsenic exposure induces changes in hippocampal morphology. Recent studies have demonstrated deficits in adult neurogenesis in the dentate gyrus of the hippocampus in both developmental and adult exposures to arsenic. Treatment for four months with 4 μg/L arsenic in drinking water reduced proliferation of neural progenitor cells and the number of mature neurons [104•]. Developmental exposure of 50 μg/L arsenic (in utero and postnatal) altered differentiation but not proliferation of neural progenitor cells in the adult hippocampus at PND63 [72]. In both studies, deficits in adult neurogenesis were ameliorated either after cessation of the use of arsenic in water [104•] or with experience in an enriched environment [72]. In vitro studies using P19 pluripotent cells cultured with varying concentrations of arsenic (7.5-75.0 μg/L) demonstrated that arsenic inhibited the formation of muscle and neuronal cells during P19 cell differentiation in a dose dependent manner. Deficits in neuronal differentiation may have resulted from reduced expression of transcription factors including *neurogenin 1*, *neurogenin 2*, and *NeuroD* as compared to control P19 cultures. Further, *Nanog* expression increased during cell differentiation, suggesting that arsenic impacts differentiation but not proliferation [105].

Altered hippocampal morphology in CA1, CA3, and the dentate gyrus was also observed during postnatal development after I.P. injection of 1, 1.5, and 2.0 mg/kg arsenic from PND4-11 [106]. Two weeks of 100 μM exposure to arsenic reduced GFAP staining in the hippocampus in another study, indicative of less neural stem cells [63], while exposure to arsenic induced deficits in cell proliferation and increased apoptosis in the brains of zebrafish [107]. These studies suggest many different types of exposures to arsenic, including brief, chronic, adult, or developmental, can impact the morphology of the hippocampus. The effect of arsenic on neurogenesis may be mediated through a signaling mechanism already discussed: altered glucocorticoid and HPA axis function. Neurogenesis is a particularly sensitive process in the brain, and as such, is prone to insults like stress activity. Recent in vitro studies done in human hippocampal progenitor cells have demonstrated biphasic responses of adult neurogenesis to cortisol; of particular interest is that increased cortisol levels inhibit proliferation and differentiation possibly mediated by the GR [108]. However, the effect of the GR on adult neurogenesis is complex, as proposed by work showing antidepressants mediate an increase in adult hippocampal neurogenesis by activating the GR [109] as well. Arsenic has an impact on adult neurogenesis and hippocampal-dependent learning and memory and induces cognitive deficits and depressive-like symptoms; altered HPA axis regulation, and particularly GR signaling in the hippocampus could underlie all of these alterations seen in arsenic-exposed animals.

### Summary

Arsenic can alter a multitude of systems in the brain. Of particular interest is HPA axis dysregulation that may underlie several behavioral deficits, particularly related to the hippocampus including alterations in adult neurogensis and Ras-MAPK/ERK signaling. Additionally, arsenic seems to have an impact on cholinergic and monoaminergic signaling, though the mechanisms are not well understood at this point. Rodent studies have provided useful corroboration of the epidemiological evidence suggesting that a number of mechanisms could underlie cognitive deficits and mood disorders observed in human populations. More research focused on the dynamics of epigenetics, particularly on mechanisms of learning and memory and mood, will be important for understanding the impact of arsenic on the brain. While it is unlikely one common unifying mechanism for arsenic’s effects will be identifed, some clues on how naturally present cations, like zinc, interact with the system, may provide insight.

## Therapeutics

Some arsenic-induced deficits have been ameliorated with therapeutics. For example, in rodent studies, deficits in adult neurogenesis can be attenuated either by abolishment of exposure to arsenic water [104•], experience in an enriched environment [72], or chronic antidepressant treatment (our unpublished observations). Reduced expression of monoamines after arsenic exposure can be reversed by treatment with curcumin [85] or taurine [102]. Studies described here have demonstrated that use of selenium, zinc, arsenic chelators, and environmental enrichment can attenuate systemic deficits due to arsenic exposure.

The use of selenium as an anti-carcinogen and chemoprotective compound and its interaction with arsenic has been extensively studied in the cancer field. Selenium forms conjugates with arsenic using glutathione for excretion [110]. A study on selenium concentrations in adults and children reported an inverse correlation between blood concentrations of selenium and urinary concentrations of arsenic [111]. In rodent studies, selenium concentrations in the cerebrum and cerebellum are reduced after exposure to 1-4 mg/L arsenic [112]. Treatment with selenium (Se) can attenuate some arsenic-induced deficits such as the inhibition of AchE in arsenic-exposed fish (*Channa punctatu)*, particularly if Se treatment precedes arsenic exposure [113]. Another naturally occurring metal, zinc, is an important component of several enzymes involved in development and CNS function. Concurrent exposure to 40 mg/kg arsenic and 4 % w/v zinc in drinking water during pregnancy induced no teratological deficits or arsenic toxicity in male offspring, as seen in arsenic-only exposure. No changes were observed in sensory reflexes, glutathione, or motor behavior, suggesting that zinc treatment attenuated arsenic-induced deficts in mice [114]. Zinc has also been shown to be protective against arsenic-induced apoptosis in neurons in vitro [115]. The mechanism by which zinc prevents arsenic-induced toxicity has not been eluciated, but we hypothesize that the Ras-MAPK/ERK pathway is involved.

In India, where efforts for intervention of arsenic-induced toxicity are at the forefront of research, the use of chelators has been suggested for attenuating the effects of chronic arsenic exposure, including thiol chelators that form insoluble complexes with arsenic [116]. One such chelator is monoisoamyl meso-2-3-dimercaptosuccinic acid (MiADMSA), a lipophilic analog of DMSA and a strong thiol chelator. Five days of MiADMSA was provided for treatment of male rats after 10 weeks of exposure to 2 mg/kg arsenic. Compared to control animals, arsenic-exposed rats displayed decreased locomotor activity, fore and hind limb strength, reduced exploration, and impaired learning in the morris water maze. Additionally, reduced levels of malondialdehyde and oxidative stress markers including superoxide dismutase, glutathione peroxidase, glutathione reductase, and glutathione transferase were measured in the cerebellum, hippocampus, and most notably, the frontal cortex. Treatment with MiADMSA attenuated all of these effects but failed to restore performance back to control levels [117].

Other notable interventions include high protein diet, antidepressants, curcumin, and exposure to enriched envrionments. Casein and pea protein, both of which have antioxidant proteins, protected against the effects of arsenic on reproductive measures in female rats [118], while curcumin in the diet attenuated the effects of arsenic on malonaldehyde, glutathione, and superoxide dismutase in several key brain regions, including decreased *TH* expression in the striatum [85]. Our own studies of treatment with fluoxetine (Prozac) or exposure to an enriched environment after developmental arsenic exposure suggests that the insults arsenic imparts can be ameliorated [72]. Research on therapeutic approaches will become more pronounced as the number of people exposed to arsenic increases. However, efforts to implement safe water programs have been successful in reducing arsenic exposure in some parts of the world [119]. Arsenic remediation endeavors remain the most effective method for reducing exposure to this insidious toxic metalloid.

## Conclusion

The most recent report provided by the Agency for Toxic Substances and Disease Registry (ATSDR) has suggested that toxic exposures to arsenic may result in memory loss and emotional instability in humans. Epidemiological studies from the past decade have provided a wealth of information supporting a strong correlation between arsenic exposure and neurological and cognitive dysfunction in children and adults. These deficits seem to be dependent on concentration, timing, and duration of exposure, with cumulative arsenic inducing more severe insults. Developmental exposure to arsenic may impart damage on critical processes involved in programming in the brain. Research in rodent models has provided sufficient evidence to suggest that arsenic toxicity affects multiple systems and specific pathways involved in several aspects of learning, memory, movement, decision making, and mood. The next phases of research will delve deeper into mechanisms of action including epigenetics and specifically focus on therapeutics for the treatment of arsenic toxicity on the brain. More information on health and exposure during the intrauterine period could be helpful in ascertaining the true impact of arsenic exposure on the central nervous system. Additionally, more epidemiological studies, particularly in the United States, would be useful in determining if the current standard (10 μg/L) will be sufficient enough to block arsenic-induced neurotoxicity, and if not, what can be done, either legislatively or pharmaceutically to alleviate arsenic-mediated cognitive deficits.
